# Peroxide of Hydrogen

**Published:** 1891-05

**Authors:** 


### Peroxide of Hydrogen.

In a paper read at the International Medical Congress at Berlin, Dr. Paul Gibier closes with the following suggestions concerning Peroxide of Hydrogen:

1. This chemical seems to have no injurious effect on animal cells.

2. It has a very energetic destructive action upon vegetable cells—microbes.

3. It has no toxic properties and is harmless when given by the mouth.

